# Second-Generation Magnesium Phosphates as Water Extractant Agents in Forward Osmosis and Subsequent Use in Hydroponics

**DOI:** 10.3390/membranes13020226

**Published:** 2023-02-13

**Authors:** Esther Mendoza, Albert Magrí, Gaëtan Blandin, Àlex Bayo, Josephine Vosse, Gianluigi Buttiglieri, Jesús Colprim, Joaquim Comas

**Affiliations:** 1 ICRA-CERCA, Catalan Institute for Water Research, Emili Grahit 101, 17003 Girona, Spain; 2 University of Girona, 17004 Girona, Spain; 3 LEQUIA, Institute of the Environment, University of Girona, Campus Montilivi, Carrer Maria Aurèlia Capmany 69, 17003 Girona, Spain

**Keywords:** forward osmosis, hydroponic culture, lettuce, nutrient solution, osmotic dilution, precipitated phosphate salt, water reuse

## Abstract

The recovery of nutrients from wastewater streams for their later use in agricultural fertilization is an interesting approach. Wastewater recovered magnesium phosphate (MgP) salts were used in a forward osmosis (FO) system as draw solution in order to extract water and to produce a nutrient solution to be used in a hydroponic system with lettuces (*Lactuca sativa*, L.). Owing to the low solubility of the MgP salts (i.e., struvite, hazenite and cattiite) in water, acid dissolution was successfully tested using citric and nitric acids to reach pH 3.0. The dilution by FO of the dissolved salts reached levels close to those needed by a hydroponic culture. Ion migration through the membrane was medium to high, and although it did not limit the dilution potential of the system, it might decrease the overall feasibility of the FO process. Functional growth of the lettuces in the hydroponic system was achieved with the three MgP salts using the recovered water as nutrient solution, once properly supplemented with nutrients with the desired concentrations. This is an innovative approach for promoting water reuse in hydroponics that benefits from the use of precipitated MgP salts as a nutrient source.

## 1. Introduction

Phosphorus (P), along with nitrogen (N) and potassium (K), is an essential nutrient in food production systems. Nowadays, P is mostly obtained from mined phosphate rock, which is a finite resource unevenly distributed around the world, so uncertainties may arise about supply [1]. The European Union (EU) has identified phosphate rock and P as two of the 27 critical raw materials of great importance to the EU economy and with a high risk associated with their supply [2]. As an alternative to mined phosphate rock, wastewater streams are renewable sources of P, and are typically locally available [3]. The recovery of P from wastewater streams (e.g., urban, industrial or agricultural wastewaters) [4,5] and its subsequent reuse, either directly or after intermediate processing, represent a major opportunity to exploit new and more sustainable pathways for the production of P fertilizers. Phosphorus has no substitute but can be reused continuously and is therefore a good example of a critical resource that can be utilized more efficiently within the circular economy framework to support sustainable growth with less pollution. Among the procedures that allow the recovery of P from wastewater streams, the chemically induced crystallization of dissolved phosphate (orthophosphate-P: H_3_PO_4_ + H_2_PO_4_^−^ + HPO_4_^2−^ + PO_4_^3−^) in the form of low soluble salts is one of the most common alternatives [6]. Precipitation is achieved by appropriately supplying metal ions to the liquid phase, frequently magnesium ions (Mg^2+^) to form magnesium phosphate (MgP) minerals. The most valued precipitated salt is struvite (magnesium-ammonium-phosphate hexahydrate, MAP, MgNH_4_PO_4_·6H_2_O) [7,8]. Nevertheless, other similar struvite-type salts can be formed in the presence of K^+^ and sodium (Na^+^), such as K-struvite (magnesium-potassium-phosphate hexahydrate, MPP, MgKPO_4_·6H_2_O), Na-struvite (magnesium-sodium-phosphate heptahydrate, MSP, MgNaPO_4_·7H_2_O), and K,Na-struvite (hazenite, Mg_2_KNa(PO_4_)_2_·14H_2_O) [9,10]. Newberyite (magnesium-hydrogen-phosphate trihydrate, MgHPO_4_·3H_2_O) and trimagnesium phosphates (bobierrite, Mg_3_(PO_4_)_2_·8H_2_O; and cattiite, Mg_3_(PO_4_)_2_·22H_2_O) may also precipitate under certain conditions [11] (Table 1).

Concerning water availability, in a recent report, the World Meteorological Organization [12] indicates that more than two billion people currently live under water stress, and that this number is expected to increase, threatening economic and social development worldwide. According to this, increasingly, water is a scarce commodity that is not given enough attention. Therefore, it is important to implement systems that allow its recovery and reuse. In this sense, forward osmosis (FO) is an interesting way to recover and purify polluted water [13,14], such as wastewater or greywater [15,16].

FO bases its extractive potential on the osmotic pressure difference between two solutions that are separated by a semi-permeable membrane, without using mechanical pressure to force permeation through the membrane [17]. The purpose is to extract water from a solution with low salinity (feed solution, FS) to a more concentrated solution (draw solution, DS). Owing to imperfect membrane rejection, during water extraction there are also ion fluxes in opposite directions (from the feed to the draw and from the draw to the feed). These fluxes represent one of the major limitations in the FO systems and entail the need for their quantification. The potential of concentrated fertilizer solutions as water extraction solution has already been tested for the recovery of water containing different contaminants [18,19,20]. In this way, the use of membranes allows the recovery of water that otherwise could not be used directly for irrigation. Different types of membrane can be used in FO. Unlike cellulose acetate membranes, thin-film composite (TFC) membranes have greater resistance to changes in the pH and temperature [21]. TFC membranes are made up mainly of two parts, the active layer (formed by a polyamide layer) and a porous layer, usually made of polysulfone to avoid mechanical stress. Recent works have explored the potential of other materials to produce FO membranes, such as chitosan [22], which is extracted from crustaceans’ shells, or with bamboo pulp [23], reaching superhigh water fluxes (>100 L/(m^2^·h)) with both membranes. One of the main causes of loss of osmotic potential in FO is the concentration polarization, which occurs mainly in the support layer due to the accumulation of salts in the porous structure or at the membrane surface [24].

In FO systems, the use of fertilizers as draw solution requires managing them dissolved. Owing to the low solubility of MgPs in water, they need to be dissolved in acidic conditions [7]. Previous experiences have already described the use of citric acid (C_6_H_8_O_7_) and nitric acid (HNO_3_) for MAP dissolution [25]. Thus, while citric acid (weak tricarboxylic acid, pK_a_ = 6.4, 4.7 and 3.1) only allows lowering the pH to values near 3.0, nitric acid (strong acid, pK_a_ = −1.4) allows reaching pH values close to 1.0. In the case of using an acid solution as extracting solution in FO, it is advisable to reach pH values not below 2.0–3.0 to preserve the membrane integrity [26].

The lack of land for cultivation, due to dedication to other uses (i.e., industrial, residential), makes hydroponic systems (soilless culture) emerge as a possible alternative. In hydroponics, the plant is in direct contact with water and nutrients. The main components of these waters are N, P and K (Table 2). The specific contents will depend on the type of crop and the applied environmental conditions [27]. This type of controlled cultivation may avoid the loss of crops due to natural events such as high temperature, prolonged periods of rain, drought and storms, allowing for a more stable production [28]. Another factor that can be controlled with hydroponic cultivation is the pollution of the soil caused by traditional crops [29]. Lettuce (*Lactuca sativa* L.) is one of the most popular leafy vegetables and it is combined with many types of food. This plant is a source of vitamin A (organic compounds with unsaturated nutritional forms), vitamin K (fat-soluble vitamins that regulate blood coagulation, bone metabolism and calcium (Ca) levels in the blood) and ascorbic acid, among others [30].

The aim of this work was to assess the use of different MgP products (struvite, hazenite and cattiite) recovered from wastewater streams as draw solution (after acid dissolution) in FO with the subsequent use of the resulting nutrient solution in a hydroponic system with lettuces. This work demonstrated, for the first time, the technical feasibility for the complete treatment line, from the recovery of MgP products [10] to their reuse in hydroponics.

## 2. Materials and Methods

### 2.1. Magnesium Phosphates Used as Draw Solution in Forward Osmosis

Three different MgP products were tested as DS (pre-acid dissolution) in a FO system. The compositional characteristics of the salts assayed are listed in Table 3, consisting of: (MgP1, S) struvite coming from the side-stream of an urban wastewater treatment plant; (MgP2, H) hazenite-type material produced from a swine denitrified effluent using newberyite particles as additive [10]; and (MgP3, C) cattiite-type material produced from a swine denitrified effluent using MgCl_2_ as additive [10]. These three MgP products were non-commercial products. Pictures of them are shown in Table S1 of the Supplementary Materials.

### 2.2. Magnesium Phosphates Dissolution Tests

A dilution ratio of 28 g MgP/L-water (7 g salt in 250 mL water) [25] was initially tested, subsequently applying a four-fold increase up to 112 g MgP/L-water (7 g salt in 62.5 mL water). Two different acids were tested to dissolve the MgP salts: citric acid (C) (4.5 N) and nitric acid (N) (5 N). Dissolution tests were carried out at an acid addition rate of 0.5 mL/min. A titration curve was plotted showing the evolution of the pH against the total amount of protons added. To verify the degree of dissolution of the MgP salts, the remaining total suspended solids (TSS) were measured once pH 3.0 was reached.

### 2.3. Forward Osmosis Dilution Tests

The FO tests were performed with commercial Aquaporin FO hollow fiber modules (mod. Aquaporin Inside^®^ HFFO6, Aquaporin A/S, Kongens Lyngby, Denmark). These modules, made with inner-selective polyamide based biomimetic active layer, had a total effective area of 0.6 m^2^. Deionized (DI) water was used as FS and acid dissolved MgP salts were used as DS. The feed and draw solutions were circulated, respectively, by the shell and bores of the HFFO6 modules (AL-DS) using two peristaltic pumps (mod. 323, Watson Marlow, Falmouth, UK). Both feed and draw solutions were circulated at 0.24 L/min. Experiments were performed with constant feed and draw recirculation speed, leading to continuous DS dilution and FS concentration. The dilution of the DS was performed in two sequential steps: (STEP 1) using 300 mL of DS (acid dissolved MgP) and 5 L of FS (DI water) and operating the system until reaching 5 L of diluted DS; and (STEP 2) using 500 mL of diluted DS produced in step 1 and 5 L of FS (DI water) and operating the system until reaching the osmotic equilibrium.

The water flux (J_w_) crossing the membrane from FS to DS was determined by measuring the increase of mass of the DS over time with a balance (mod. PCB 6000-1, Kern, Balingen, Germany). The evolution of the salt content in the FS was determined with an electrical conductivity meter (Crison Instruments SA, Alella, Spain) according to a NaCl-conductivity calibration curve, which was used to calculate the reverse salt flux (J_s_) [13]. All data were recorded using a Bluetooth-based system provided by Instrument Works (Waterloo, Australia) as in former studies [35,36]. Samples of DS at the beginning and at the end of each dilution step were used to assess ion migration across the membrane (from DS to FS).

### 2.4. Hydroponic System

#### 2.4.1. Experimental Setup and Procedure

A hydroponic system was built with NFT (i.e., nutrient film technique) PVC channels and equipped with four fluorescent LED tubes of 120 cm length (cold white and blue + red, 18W, Osram, Munich, Germany) that were placed 60 cm above the channels. Light cycles of 14 h ON and 10 h OFF were performed to mimic the daily cycle of natural light. Sensors for temperature, relative humidity (mod. Hobo Pendant^®^ U23-001A, Onset, Bourne, USA), and light intensity (mod. Hobo Pendant^®^ UA-002-64, Onset, Bourne, USA) allowed data to be recorded recording at 30-min intervals to monitor the environmental conditions. Lettuce planters were bought in a local market, rinsed to remove the soil, and introduced into the hydroponic system.

Four hydroponic channels were used to test three different nutrient solutions containing dissolved MgP salts, plus one control per experimental cycle. Each channel fitted eight plant pots (distance: 8 cm) filled with inert expanded clay aggregates to support the plants’ root system. Two experimental cycles were conducted lasting three weeks each. Weekly, the nutrient solutions were renewed, the number of leaves of the lettuces was recorded and the dry leaves were removed.

To analyze the plant growth and health, three lettuces were selected on the first day of planting and one representative lettuce of average growth was selected per each tested condition on the last day of the test. These lettuces were cleaned with DI water, measured, and dismembered according to their functional parts (i.e., leaves, roots, and shoots). Leaf area was determined using “Easy Leaf Area Free” mobile phone application (last updated 31 July 2015) developed by Easlom and Bloom [37]. Fresh and dry weight (after oven drying at 70 °C for 48 h) [38] were recorded. Concerning nutrient solutions, the final volume, pH, and electrical conductivity were measured weekly. Moreover, samples of the influent water were taken to determine their composition by ion chromatography.

#### 2.4.2. Nutrient Solutions for Hydroponics

The composition of the nutrient solutions obtained through FO depended on the used MgP salt, the applied acidifying agent, and the achieved dilution rates, as will now be discussed. For the correct growth of lettuces, the NPK content in the nutrient solution should be approximately within the reference ranges listed in Table 2. In addition, the excess or deficit of certain ions may be critical for some crops.

The nutrient solutions obtained through FO were tested in two hydroponic experimental cycles planned as follows (Table 4):

- Experimental cycle no. 1. (1) Commercial fertilizing solution (control) made up of NH_4_H_2_PO_4_ + KNO_3_ + Ca(NO_3_)_2_ + MgSO_4_; (2) hazenite dissolved with citric acid (HC); (3) hazenite dissolved with nitric acid (HN); and (4) hazenite dissolved with nitric acid and supplemented with KNO_3_ (1M) to reach NPK levels similar to those of the fertilizing solution (HN+).
- Experimental cycle no. 2. (1) Control; (2) struvite dissolved with nitric acid and supplemented with KNO_3_ (SN+); (3) HN+; (4) cattiite dissolved with nitric acid and supplemented with KNO_3_ (CN+).

Additionally, a micronutrient solution containing Cu, Fe, MnSO₄, ZnSO_4_, H_3_BO_3_ and (NH_4_)_6_Mo_7_O_24_ was added to all the solutions.

In the experimental cycle no. 1, hazenite-derived solutions were chosen since the P concentration was the closest to the reference values (Table 2) and this salt also contributed to the supply of K. During the first cycle, the control condition finished the water in the channel before the scheduled weekly water change in weeks 2 and 3. The same happened in condition HN+ in week 2. Even though more of the respective solution was added to not let the plants dry out, plants were visibly affected, which is why control and HN+ conditions were repeated in the second cycle of the experiment.

In the experimental cycle no. 2, nitric acid dissolved solutions of the three MgP salts considered in this study were tested once supplemented with KNO_3_. The salts dissolved in nitric acid were preferred over those in citric acid since they had a contribution of nitrate, one of the main nutrients for plants.

### 2.5. Analytical Methods

Precipitated salts were analyzed using X-ray diffraction (XRD) and the total content of the main constituents (Na, K, Ca, Mg, and P) was measured after microwave + HNO_3_/H_2_O_2_ digestion using inductively coupled plasma-optical emission spectrometry (ICP-OES) (mod. 5100, Agilent Technologies, Santa Clara, USA). Total N was determined by elemental analysis (mod. 2400 Series II Elemental Analyzer, Perkin Elmer, Waltham, USA).

Water samples were analyzed according to APHA et al. [39]. The pH value was measured with a pH-meter (mod. sensION+ PH3, Hach, Düsseldorf, Germany) and the electrical conductivity was measured with a conductivity meter (mod. EC-Meter Basic 30+, Crison Instruments SA, Alella, Spain). Total suspended solids (TSS) were measured gravimetrically after sample filtration with a glass microfiber filter and subsequent drying to constant weight. The concentration of the soluble cations (i.e., ammonium (NH_4_^+^), sodium (Na^+^), potassium (K^+^), magnesium (Mg^2+^), and calcium (Ca^2+^)), as well as the concentration of the soluble anions (i.e., nitrite (NO_2_^−^), nitrate (NO_3_^−^), chloride (Cl^−^), sulfate (SO_4_^2−^), and phosphate (PO_4_^3−^)), was determined by ion chromatography (mod. ICS-5000, Dionex, Sunnyvale, USA) after filtering samples with 0.2-μm nylon filters.

### 2.6. Calculations

For the FO tests, the water flux (J_w_) in L/(m^2^·h) was calculated with the variation of the DS mass along time, as follows (Equation (1)):

(1) $$J_{w}=\frac{\Delta m_{DS}}{A\cdot \rho \cdot \Delta t}$$

where Δm_DS_ is the DS mass increase over time (kg), Δt is the time variation (h), A is the membrane area (0.6 m^2^) and ρ is the water density (1 kg/L).

Reverse salt flux (J_s_) in g/(m^2^·h) was calculated based on the FS conductivity (Equation (2)):

(2) $$J_{s}=\frac{C_{FS,f}\cdot V_{FS,f}-C_{FS,0}\cdot V_{FS,0}}{A\cdot \Delta t}\;$$

where C_FS,0_ and C_FS,f_ represent initial and final salt concentration (g/L) in FS –NaCl–, respectively, and V_FS,0_ and V_FS,f_ represent initial and final FS volume (L), respectively.

Total ion migration (i.e., the percentage of ions that moved from the DS towards the FS by the end of the experiment in relation to the ion content in the initial DS in the first dilution step) was calculated considering the ratio between theoretical and measured ion concentrations in final DS, as follows (Equation (3)):

(3) $$total\;ion\;migration\;\left(\%\right)=\left(1-\frac{C_{DS,f{\_}_{2}}\cdot \;d}{C_{DS,0\_1}}\right)\cdot 100$$

where: C_DS,0_1_ and C_DS,f_2_ refer to individual ion concentrations in the DS at the beginning and the end of the dilution process (mg/L), respectively; i.e., the initial concentration in the first dilution step and the final concentration in the second dilution step, respectively. d is the total dilution factor, which is the product of the first dilution by the second.

Growth of the lettuce plants was assessed by considering number of produced leaves, leaf area, fresh and dry weight, as well as growth parameters commonly used elsewhere [38,40,41], and according to the formulas listed below:

(4) $$RGR=\frac{\ln W_{2}-\ln W_{1}}{t_{2}-t_{1}}$$

(5) $$SLA=\frac{\left(LA_{2}/LW_{2}\right)-\left(LA_{1}/LW_{1}\right)}{2}$$

where: W is the total dry weight of the plant (g), t is time, LA is the leaf area (cm^2^) and LW is the dry weight of the leaves (g). t_1_ and t_2_ (days) refer to the day of planting and harvesting of each plant, respectively. The relative growth ratio (RGR, g/(g·day)) (Equation (4)) allows knowing the growth rate of a plant regardless of its size. The specific leaf area (SLA, cm^2^/g) (Equation (5)) indicates the robustness and/or density of the leaves.

## 3. Results and Discussion

### 3.1. Acid Dissolution of the Magnesium Phosphates

**Figure 1:**
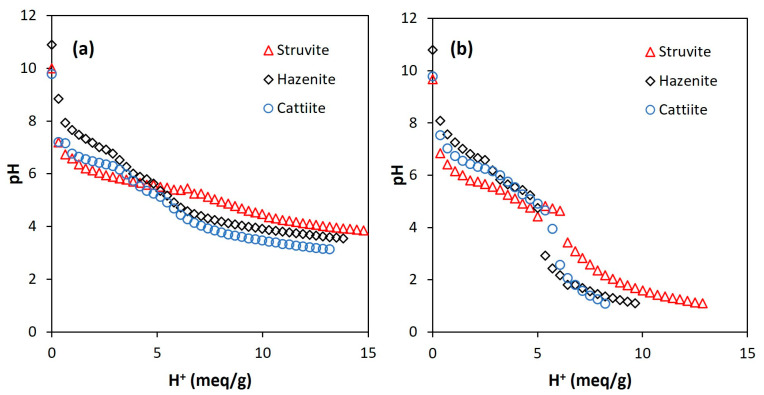
Titration curves for the acid dissolution of the MgP salts (struvite, hazenite and cattiite) using citric acid (**a**) and nitric acid (**b**). Dilution ratio: 28 g salt per liter of water.

The acid dissolution of the three MgP salts (i.e., struvite, hazenite and cattiite) led to similar titration curves depending on the acid used. Figure 1 shows such patterns when considering 28 g/L as the dilution ratio. In the case of using citric acid, the titration curves did not show abrupt changes in the pH value. The slowest pH decrease rate was measured for struvite, which could be caused by the nature of the salt (i.e., the ammonium released behaved like a pH buffer; this was the least hydrated salt). The use of nitric acid did not imply big differences between salts either, struvite again being the salt that offered the most resistance to decreasing the pH. Unlike the previous case, a sharp drop occurred at pH 5.5–3.0, making it difficult to measure a stable pH-value within this range.

Under the dilution ratio of 112 g/L, MgP salts showed good capacity of dissolution at pH 3.0 (data not shown). Thus, undissolved TSS reached 2.5% of the initial solids content as a maximum (Table S1 in Supplementary Materials), confirming the low loss of salts (not dissolved) occurring during the dissolution process. Struvite needed the largest amount of acid for dissolution. For this salt, final TSS analysis only revealed 1.5–1.8% of solids loss. According to these results, almost complete dissolution of the MgP was obtained for all the conditions tested.

### 3.2. Water Extraction and Nutrients Dilution with Forward Osmosis

#### 3.2.1. Forward Osmosis Dilution Potential

**Figure 2:**
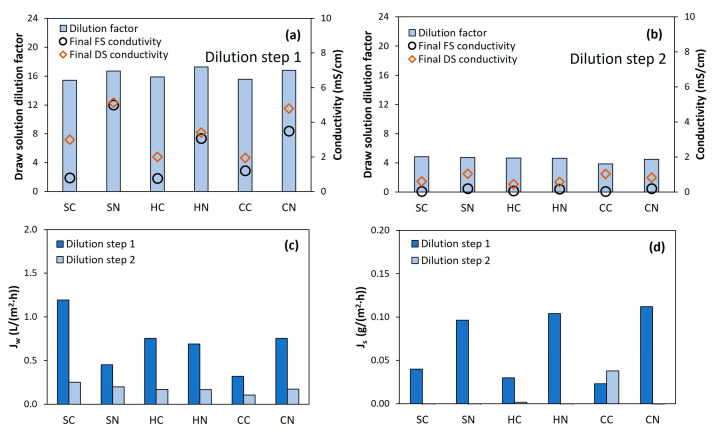
Results for the FO dilution tests. DS dilution factors in step 1 and step 2 of the FO process (**a**,**b**), water flux through the membrane (**c**) and reverse salt flux through the membrane (**d**). Reference for MgP salts: S, struvite; H, hazenite; C, cattiite. Reference for acids: C, citric acid; N, nitric acid.

The FO dilution tests were performed in two sequential steps. In the first step, high DS dilution was reached, equivalent to a dilution factor of about 16 times (Figure 2a). In the second step, an additional dilution factor of around four times was achieved (Figure 2b), leading to an overall dilution factor of above 60 times (Figure S1 in Supplementary Materials). By the end of the second dilution step, DS and FS conductivities reached similar values (<1.5 mS/cm), attesting that the system had nearly reached the osmotic equilibrium, and that no more water could be extracted with the nutrient solution. In fact, when looking at the water permeation flux (Figure 2c and Figure S2 in Supplementary Materials), low values were reached even during the first dilution step. This fact can be explained by the rapid dilution of the concentrated DS. Consequently, the DS partly lost the osmotic potential during the first hours of the FO process, leading to low permeation flux, so the overall filtration time took longer than 25 h to reach the targeted volume (Figure S2 in Supplementary Materials). Thus, such long filtration time was inherent to the setup design; from the industrial scale-up point of view, it may result in high membrane area requirements to achieve the proposed dilution in less time.

Interestingly, very low reverse salt fluxes were observed from the DS to the FS (Figure 2d), much lower than in other studies with similar FO membranes [19,42]. These results indicate that most of the ions from the initial DS seemed to remain in the original solution and, thus, they were part of the nutrient solution usable in hydroponics. Comparatively, the MgPs acidified with nitric acid exhibited slightly higher reverse salt flux than when using citric acid. Such behavior could be related to the fact that the nitrate ion is smaller than the citrate ion and so it is more prone to diffuse through the FO membrane [43].

#### 3.2.2. Total Ion Migration through the Forward Osmosis Membrane

**Figure 3:**
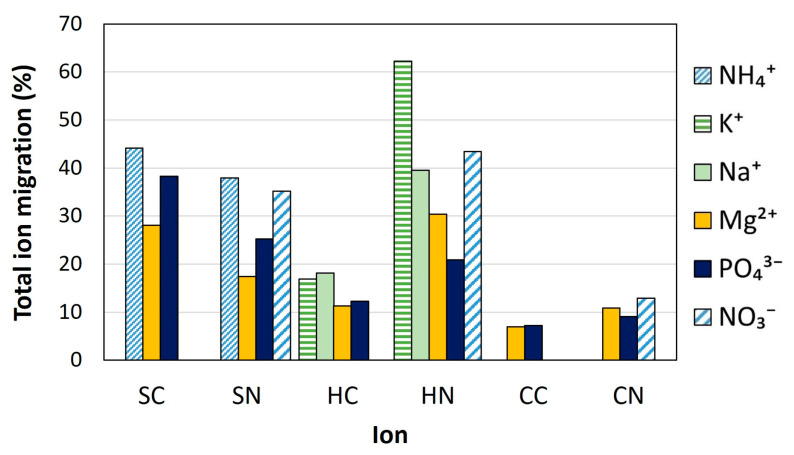
Total ion migration through the membrane (% ions lost to FS in the two dilution steps). Reference for MgP salts: S, struvite; H, hazenite; C, cattiite. Reference for acids: C, citric acid; N, nitric acid. Reference for acids: C, citric acid; N, nitric acid.

Ion migration from the DS to the FS through the FO membrane is not desirable since it implies loss of valuable nutrients. Thus, ion migration should be kept to a minimum for an efficient FO performance. Overall, in this study, even if calculated reverse fluxes were low, medium to high ion migration was observed for all the tests and ions (Figure 3). Monovalent ions (i.e., NH_4_^+^, Na^+^, K^+^ and NO_3_^−^) migrated to the FS to a greater extent than divalent (Mg^2+^) and eventually trivalent (PO_4_^3−^) ions. This behavior is attributable to the smaller hydrated radius and the lower electrostatic repulsions with the membrane of the monovalent ions, which passed across the membrane more easily to balance the osmotic pressure between the two solutions. Cation migration was favored by the negatively charged surface of the membrane [44,45], while NO_3_^−^ migration could be explained because of the diffusion mechanism, which would imply the transfer of this anion through the membrane to balance the positive charges. This behavior was clearly observed in the tests with hazenite, where Na^+^ and K^+^ migration was much higher with nitric acid than with citric acid (Figure 3). This higher migration was due to the high diffusivity of Na^+^, K^+^ [46], and NO_3_^−^ [47], with the latter one passing through the membrane in similar proportions than cations to balance the positive charges (solution diffusion mechanism). Otherwise, Na^+^ and K^+^ migration with citric acid was lower since there was not a counter ion (i.e., NO_3_^−^) able to diffuse through the membrane. For the tests with struvite, high NH_4_^+^ migration was also found regardless the acid applied, leading also to the highest phosphate migration. In that case, NH_4_^+^ migration could be explained by the smaller hydrated radius than other cations, which would make it pass through the membrane more easily. The higher Mg^2+^ and phosphate migration observed might be explained by the higher ion contents at the initial DS (struvite was the least hydrated phosphate tested salt). In the case of cattiite, ion migration was lower than for the other MgP minerals (Figure 3) as cattiite only contains Mg^2+^ and phosphate, which are not monovalent, so with a scarcer diffusion through the membrane [48]. These results point out the complexity of the ion transport in FO and the need to mitigate these ion fluxes. The length of batch-operated experiments could also cause significant ion losses, resulting in numerous passages of solutions across the membrane. In this sense, another study showed that, in FO, there is less diffusion when a system is designed to allow continuous operation or with a shorter operating time [49].

#### 3.2.3. Composition of the Diluted Draw Solution for Its Application in Hydroponics

The choice of the best diluted DS as nutrient solution in view of its further application in the hydroponic culture of lettuce depends on different factors, such as FO performance and nutrient composition and concentration. Concerning FO performance, tests with cattiite showed the lowest ion migration (Figure 3), which means less losses of ions to the FS and, thus, more efficient performance. However, cattiite only contains one macronutrient (P) and one mesonutrient (Mg). Struvite tests showed higher nutrient losses, but struvite also contains N, which reduces the need for an external supply of this nutrient. Although hazenite contains K, one of the main nutrients for plants, it also contains Na, which might be toxic at high concentrations. Na concentration in the nutrient solutions assayed reached up to 32 mg/L, whilst it is not recommended to exceed 150 mg Na^+^/L, especially when there is Cl^−^ in the solution [27]. Na^+^ migration to FS may be considered as an advantage, since lower Na^+^ content will be present in the final DS for use in hydroponics. Regarding the acid used to dissolve the mineral salts, even though in general terms more nutrients were lost using nitric acid, the presence of NO_3_^−^ in the final DS is an advantage since NO_3_^−^ is one of the main nutrients for plant growth.

**Figure 4:**
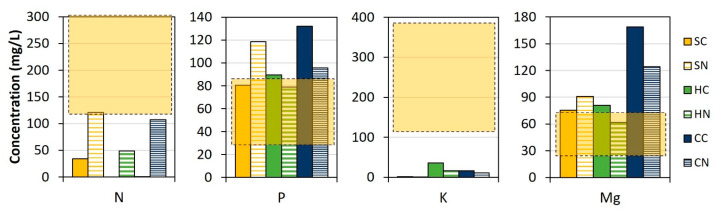
Nutrient concentration after DS dilution by FO in view of its further application in hydroponics and estimated optimal ranges (bars, ±30% values from Table 2) with the optimal ranges (yellow squares). Reference for MgP salts: S, struvite; H, hazenite; C, cattiite. Reference for acids: C, citric acid; N, nitric acid.

Concerning the obtained dilution levels, it is important to attain a proper nutrient content for hydroponics (Table 2), i.e., within desired concentration ranges. Low nutrient concentrations will lead to a poor plant growth, but high concentrations might result in plant toxicity. Therefore, optimal composition was selected for those cases in which final nutrient concentrations were within or below the desired ranges for lettuce growth (Figure 4), solving imbalances by adding a nutrient supplement without posing risk to the plant health. HN reached Mg and P concentrations within the required ranges at the end of the DS dilution and also had some K (Figure 4), and thus was selected as the best candidate for further application in hydroponics. HN was followed by SC, although in this case Mg and P concentrations were slightly above the desired ranges. In the tests using struvite (SC and SN), NH_4_^+^-N concentrations in the diluted DS (34 mg/L for SC and 39 mg/L for SN) were not far from the optimum concentration found in the commercial nutrient solution (about 25 mg/L). The other tested conditions led to higher P and Mg contents than desired (Figure 4) but reaching a slightly higher DS dilution would result in P and Mg concentrations within the appropriate ranges. 

Nonetheless, it is not common to achieve such low nutrient concentrations in FO and, thus, the achieved concentrations can be considered as satisfactory. Most of the studies found in the literature, in fact, point out the need to further dilute the DS to be able to apply it in hydroponics [50,51,52]. Proper DS dilutions with FO were only achieved after applying pressure [53,54], or with higher nutrient losses to the FS [13].

### 3.3. Hydroponic System

#### 3.3.1. Experimental Conditions

The experiment was conducted in two cycles of three weeks each, with about 3 °C higher temperature in the second cycle (Cycle 1: 24 ± 2 °C; Cycle 2: 27 ± 2 °C), while the light intensity (avg. 4000 ± 1400 lux due to light gradient in the system) and relative humidity (avg. 64 ± 8%) were similar in both experiments. The average N, P, K and Mg concentrations in the nutritive solutions applied in the tested conditions with the ideal ranges are shown in Figure S3. Due to the lacking or low concentrations of some of the main nutrients in the diluted solution from FO, KNO_3_ was added as a supplement for the conditions HN+, SN+ and CN+ to reach values in accordance with those found in literature (Table 2).

#### 3.3.2. Plant Growth Analysis

Plants grown in the control condition with commercial nutrient solution showed different growth in both cycles (four more leaves produced in the first cycle but almost 40% higher leaf area produced in the second cycle). This could be due to the rather higher temperature in cycle 2 (where temperatures closer to 20 °C are preferrable for lettuce growth [41,55]) and the fact that the initial plants for each cycle were noticably different in size (e.g., plants had in avg. eight and five leaves at the beginning of cycles 1 and 2, respectively). Consequently, the growth parameters of the conditions tested in the different cycles are compared to their respective control condition and subsequently with each other.

**Figure 5:**
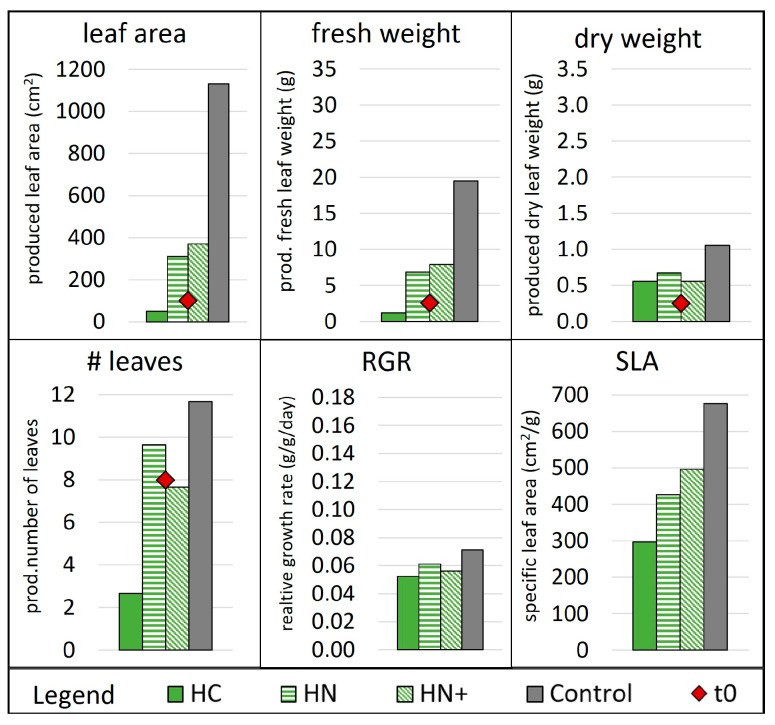
Plant growth parameters in week 3 of cycle 1. Number of leaves, leaf area, fresh and dry weight at t0 are indicated as red dots while the additionally produced quantities after 21 days are indicated as bars. Abbreviations: RGR, relative growth rate; SLA, specific leaf area. Reference for MgP salts: H, hazenite. Reference for acids: C, citric acid; N, nitric acid. +, supplemented with KNO_3_.

The first cycle included HC, HN, HN+ conditions and control (Figure 5). Condition HC failed to grow lettuces, which may be explained by the lack of nutrients in the solution, with very low N and K concentrations (Figure S3). The control grew about twice as much in terms of produced weight and three times in terms of produced leaf area as both the HN and HN+ conditions (Figure 5), even while frequently finishing the water before the scheduled time. Conditions HN and HN+ performed similarly (RGR of 0.056 & 0.061 g/(g·day), Figure 5), producing fewer leaves that were smaller in size but a little thicker (lower SLA, Figure 5) than control leaves. The results indicate that even if nutrient supply in HN was below the ideal range for hydroponics, the growth of the plants was similar, in cycle 1, to HN+ condition with extra nutrient supply (see picture in Figure S4). However, control plants and HN+ plants that ran out of water at least once were visibly affected by this incidence, which is noticeable also in the rather low RGR (highest RGR in control with 0.071 g/(g·day)) when compared with the literature values e.g., 0.08 (at 10 °C) to 0.14 g/(g·day) (at 20°C) [41] or 0.113 (mean) with −0.036 & 0.295 g/(g·day) (min & max) [56]. As a result, HN+ and control conditions were repeated in the second cycle to confirm wheter the lack of water affected plant growth.

**Figure 6:**
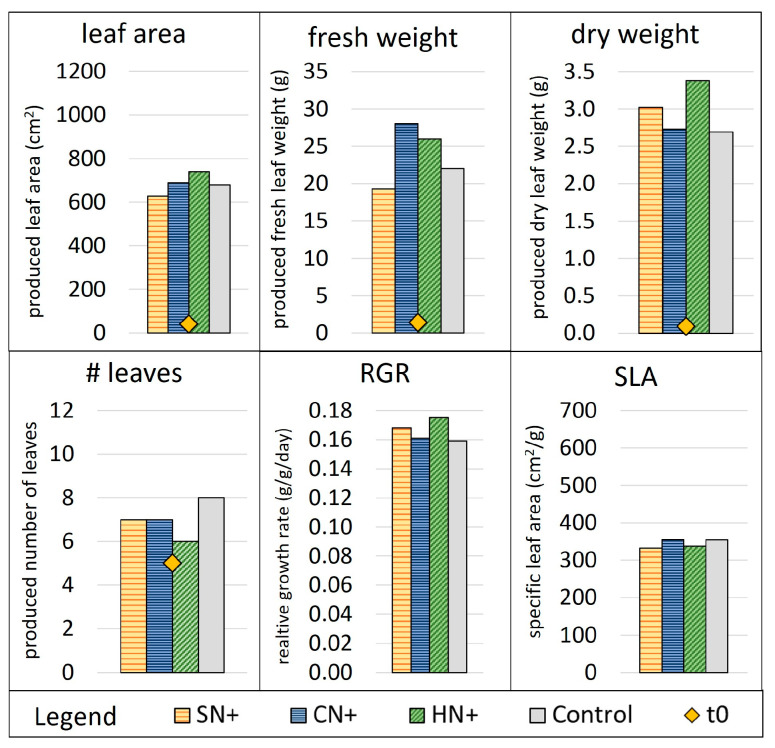
Plant growth parameters in week 3 of cycle 2. Number of leaves, leaf area, fresh and dry weight at t0 are indicated as yellow dots while the additionally produced quantities after 21 days are indicated as bars. Abbreviations: RGR, relative growth rate; SLA, specific leaf area. Reference for MgP salts: H, hazenite. Reference for acids: C, citric acid; N, nitric acid. +, supplemented with KNO_3_.

In the second cycle (SN+, CN+, the repeated HN+, and control) the initial (t0) plants had on average of three leaves and 63% dry mass less than in the first cycle. Nevertheless, plants of all conditions produced higher dry and fresh weight as well as higher leaf area (Figure 6), while growing fewer leaves than cycle 1 (excluding HC, Figure 5). This is also displayed in the observably higher magnitude for RGR of cycle 2 compared with cycle 1 (Figure 5 and Figure 6), as well as RGR of the previously mentioned literature [41,56]. The plants of CN+, SN+ and the control in cycle 2 grew big leaves of increasingly less stable structure along the weeks. The leaves had visually weaker leave blades with elongated and proportionally thin stems and petioles, despite the lower SLA indicating an already higher thickness (dry mass per area) of leaves compared to previous cycle (see picture in Figure S4). An exception to this was condition HN+, which continuously had strong petioles and were stable in structure throughout the leaves, which is surprising since this condition produced the lowest number of leaves but the highest leaf area up to this point. This could be explained by HN+ also having produced the highest dry mass at the same time. On the other hand, SN+ plants showed the optically weakest structure, despite performing similarly to HN+ regarding plant growth parameters, potentially due to the phosphate concentration (118 mg/L), which was higher than the ideal range (30–80 mg/L), which could be toxic to the plants. The water in HN+ briefly ran out again in the third week, but no noticeable effect was observed in this cycle. Conversely, due to the noticably higher RGR of HN+ in the second cycle than in the first one, it can be concluded that the low growth rate of cycle 1 control and HN+ could be related to the drying out, subsequently disproving the prior conclusion in cycle 1 that HN, which did not face the same issue, acheived a comparable growth rate to HN+.

Overall, all experimental conditions in cycle 2 produced similar or higher dry weight than their control plants (Figure 6), even while growing slightly fewer leaves. The same is observed for for the leaf area and fresh weight, with only SN+ performing slightly worse than the control. However, in RGR, all conditions (cycle 2) performed equally as well or slightly better than the control (Figure 6).

Finally, plants grown in all experimental conditions and the control plants were similar in color, though some necrotic edges (i.e., tipburn) were observed in HN+ plants (see picture in Figure S4). The tipburn is usually caused by calcium deficiency, the concentration of which in solution was minimal (data not shown) and it increases with growth rate [57], which can explain why this symptom only appeared in the bigger plants of HN+ condition. Additionally, tipburn could be caused by stress generated by temperatures over 25 °C [58]. These two factors were present in the system and could, therefore, induce tipburn in the lettuces. A more balanced nutrient solution, also including the mesonutrients calcium and sulphate, and a cooler environment should not have induced this symptom in the lettuces.

The described results show that diluted MgP solutions were suitable to grow lettuces in hydroponic cultures. However, only those conditions with KNO_3_ supplement showed a comparable growth with the controls. Even if at the end of cycle 2, some plants showed tipburn; this could have been caused by the experimental conditions and by the plants being too close to each other. Additionally, Na^+^, which might be toxic for the plants, but is present in hazenite, did not seem to be dangerous for the growth of the plants, since HN+ condition had the plants that performed best, in both cycles. Overall, these are successful results that open the door to decrease the demand of industrially produced P while promoting the valorization of second-generation P.

## 4. Conclusions

An innovative approach was evaluated as a proof of concept for the use of MgP salts as DS in FO in order to extract water and produce a nutrient solution to be used subsequently in a hydroponic system with lettuces. The main conclusions reached are as follows:

- Wastewater-precipitated MgP salts, such as struvite, hazenite and cattiite were almost completely dissolved in water (at dissolution ratios from 28 to 112 g mineral per liter of water) using citric and nitric acids when final pH was set to 3.0.
- FO allowed reaching a dilution level of the DS close to that required for hydroponics and no further dilution was needed. Ion migration across the membrane (from DS to FS) was not limiting since the desired dilution was achieved. Ion migration tended to compensate the charges, involving preferential pairs such as K^+^-Cl^−^-Na^+^, K^+^-NO_3_^−^, and NH_4_^+^-NO_3_^−^. Even if reverse fluxes were low, ion migration (which is translated in nutrient losses) was medium to high, especially for monovalent ions, which decreases the economic efficiency and feasibility of the FO technology. In this sense, more selective membranes or different DS are required to reduce these fluxes. Considering the target of FO, it could be interesting to dissolve the MgP salts with sulfuric acid, since it is a divalent ion, which will decrease the migration of other ions through the membrane compared with nitric acid, and at the same time the sulphate can be used by plants, since it is a mesonutrient.
- Functional growth of lettuces in a hydroponic system was achieved with the water recovered using FO. The tested conditions with MgP salts supplemented with KNO_3_ produced plants of comparable weight and leaf area as the control condition, with HN+ being the most stable and having the biggest plants, even when compared to the respective control condition. The Na content in hazenite was shown not to be a problem for plant development. The tested MgP salts were proved as an accurate nutrient supply for plant growth, making these by-products valuable fertilizers.

This study was a first proof of concept, moving towards application using real streams. Other challenges such as fouling and limited dilution rate may be observed. Thus, future studies should focus on testing real wastewater streams as feed solution and increasing the efficiency of the FO process by improving water fluxes while reducing reverse salt fluxes.

## Figures and Tables

**Table 1:** Magnesium phosphate (MgP) minerals formable in wastewater crystallization processes.

Name	Empirical Formula	Molecular Weight (g/mol)	P Content (wt%)	Mg/P Molar Ratio
Struvite (magnesium ammonium phosphate, MAP)	MgNH_4_PO_4_·6H_2_O	245	12.6	1.00
K-struvite (magnesium potassium phosphate, MPP)	MgKPO_4_·6H_2_O	266	11.6	1.00
Na-struvite (magnesium sodium phosphate, MSP)	MgNaPO_4_·7H_2_O	268	11.5	1.00
K,Na-struvite (hazenite)	Mg_2_KNa(PO_4_)_2_·14H_2_O	553	11.2	1.00
Newberyite (magnesium hydrogen phosphate trihydrate)	MgHPO_4_·3H_2_O	174	17.8	1.00
Bobierrite (trimagnesium phosphate octahydrate)	Mg_3_(PO_4_)_2_·8H_2_O	407	15.2	1.50
Cattiite (trimagnesium phosphate twenty-two hydrate)	Mg_3_(PO_4_)_2_·22H_2_O	659	9.4	1.50

**Table 2:** Concentration of NPK (mg/L) in standard nutrient solutions for hydroponics, according to previous studies.

Macronutrients			Mesonutrients			
N	P	K	Mg	Ca	S	Reference
210	31	234	34	160	64	[31]
168	41	156	36	160	48	[32]
200–236	60	300	50	170–185	68	[33]
168	31	273	48	180	336	[34]

**Table 3:** Main compositional characteristics of the magnesium phosphate (MgP) products used as draw solution (DS) in the forward osmosis (FO) system.

Ref.	XRD—Dominant Mineral Phase	EA & ICP—Composition (wt%)					
		N	P	K	Ca	Mg	Na
S (MgP1)	Struvite	5.3	11.5	0.0	0.9	9.4	0.2
H (MgP2)	Hazenite (w/Newberyite)	0.7	17.1	8.0	0.4	11.7	6.0
C (MgP3)	Cattiite	0.0	10.9	0.1	1.1	10.0	0.1

EA: Elemental Analysis; ICP: Inductively Coupled Plasma; XRD: X-ray diffraction.

**Table 4:** NPK content (mg/L) in the nutrient solutions used in the hydroponic culture of lettuces.

	Experimental Cycle No. 1				Experimental Cycle No. 2			
	Control	HC	HN	HN+	Control	SN+	HN+	CN+
NH_4_^+^-N	23	0	0	0	25	43	0	0
NO_3_^−^-N	168	0	33	105	156	145	124	153
PO_4_^3−^-P	36	83	76	67	37	118	66	89
K^+^	187	41	20	153	189	182	250	202
Mg^2+^	38	81	68	57	38	85	56	116

Reference for MgP salts: S, struvite; H, hazenite; C, cattiite. Reference for acids: C, citric acid; N, nitric acid. +, supplemented with KNO_3_.

## Data Availability

Not applicable.

## Supplementary Materials

The following supporting information can be downloaded at: https://www.mdpi.com/article/10.3390/membranes13020226/s1. Table S1: View of the MgP products used as DS in FO. Table S2: TSS not dissolved by the end of the acid dissolution test. Figure S1: Total dilution factor in the 2-step FO. Figure S2: Filtration kinetics example. Figure S3: Nutrient solutions tested in the hydroponic experiments. Figure S4: Pictures of lettuce plants.
