# Supplementary material for: Second-Generation Magnesium Phosphates as Water Extractant Agents in Forward Osmosis and Subsequent Use in Hydroponics
Source: Membranes (Basel). 2023 Feb 13;13(2):226. doi: 10.3390/membranes13020226 (PMC9961049; doi:10.3390/membranes13020226)
Supplement: Supplementary file 1 [file membranes-13-00226-s001.zip › membranes-2198937-supplementary.pdf]

membranes

Supplementary material

Second-Generation Magnesium Phosphates as Water Extractant Agents in Forward Osmosis and Subsequent Use in Hydroponics

Esther Mendoza [1,2,*,†], Albert Magrí [3,*,†], Gaëtan Blandin [3], Àlex Bayo [3], Josephine Vosse [1,2], Gianluigi Buttiglieri [1,2], Jesús Colprim [3] and Joaquim Comas [1,3]

[1] ICRA-CERCA, Catalan Institute for Water Research, Emili Grahit 101, 17003 Girona, Spain
[2] University of Girona, Spain
[3] LEQUIA, Institute of the Environment, University of Girona, Campus Montilivi, Carrer Maria Aurèlia Capmany 69, 17003 Girona, Spain
* Correspondence: emendoza@icra.cat (E.M.); albert.magri@udg.edu (A.M.)
† These authors contributed equally to this paper.

2. Materials and Methods

2.1. Magnesium Phosphates Used as Draw Solution in Forward Osmosis

Table S1. View of the magnesium phosphate (MgP) products used as draw solution in forward osmosis (FO).

**Ref.**	**MgP1**	**MgP2**	**MgP3**
XRD - Dominant mineral phase	Struvite	Hazenite (w/ Newberyite)	Cattiite
XRD diffractograms *	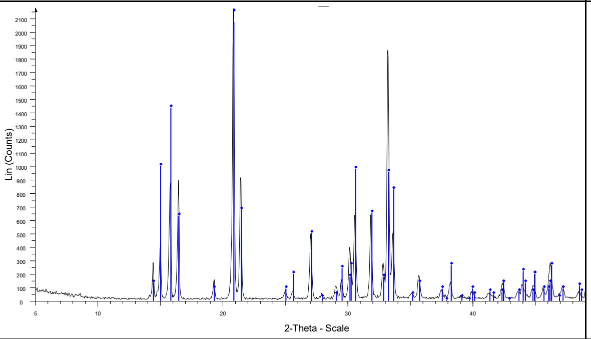	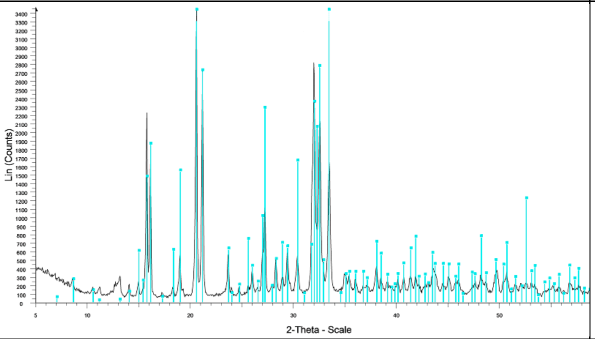	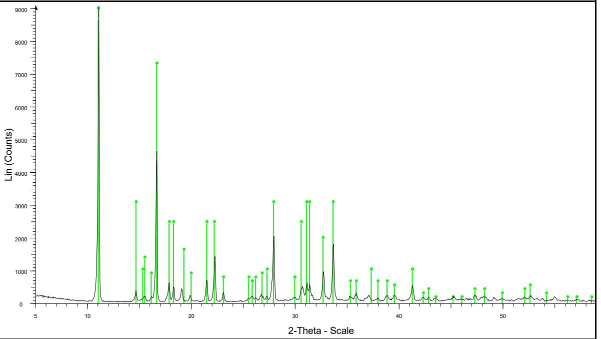
View of the mineral phase	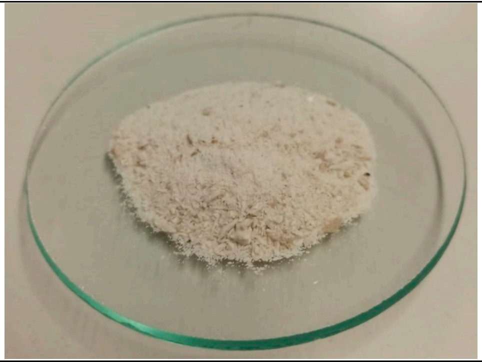	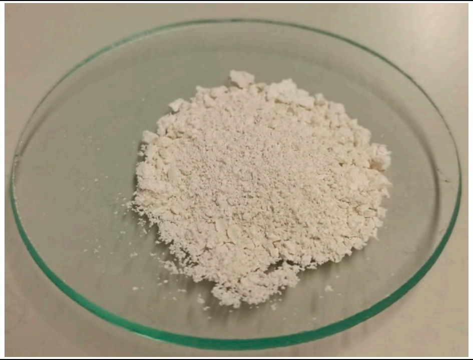	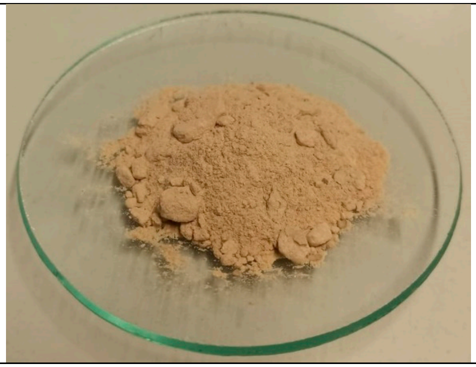

* XRD diffractograms for hazenite and cattiite were previously published in Company, E.; Farrés, M.; Colprim, J.; Magrí, A. Exploring the recovery of potassium-rich struvite after a nitrification-denitrification process in pig slurry treatment. *Sci. Total Environ.* **2022**, *847*, 157574.

3. Results and Discussion

3.1. Acid Dissolution of the Magnesium Phosphates

Table S2. TSS content after acid dissolution of the MgP salts (pH 3.0) if considering 112 g salt per liter of water as the initial dilution ratio.

Reference	% TSS final vs. initial solids content
SC	1.8
SN	1.5
HC	1.5
HN	1.2
CC	1.4
CN	2.5

Reference for MgP salts: S, struvite; H, hazenite; C, cattiite.
Reference for acids: C, citric acid; N, nitric acid.

3.2. Water Extraction and Nutrients Dilution through Forward Osmosis

3.2.1. Forward Osmosis Dilution Potential

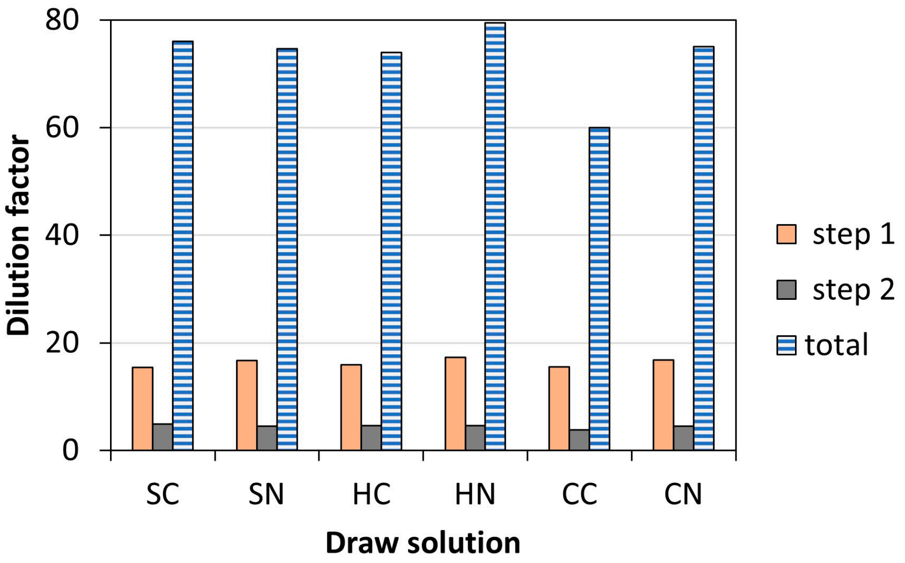

Figure S1. Total dilution factor achieved for the different draw solutions used in the 2-step forward osmosis (FO) process. Reference for MgP salts: S, struvite; H, hazenite; C, cattiite. Reference for acids: C, citric acid; N, nitric acid.

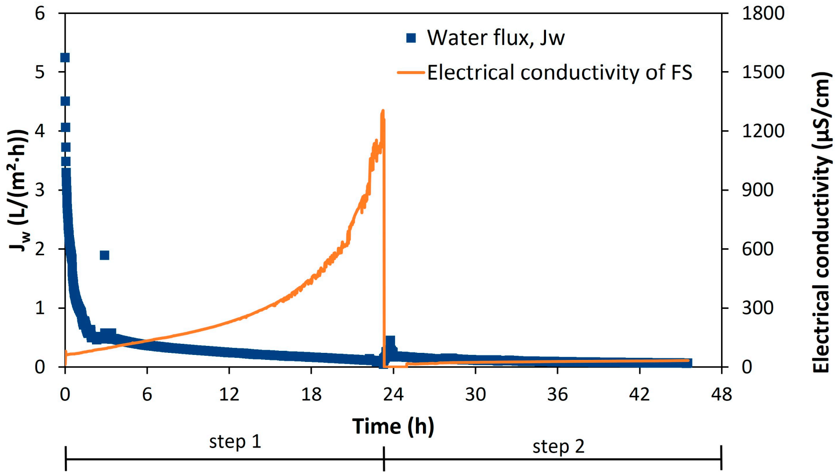

Figure S2. Filtration kinetics example (CC).

3.3. Hydroponic System

3.3.1. Experimental Conditions

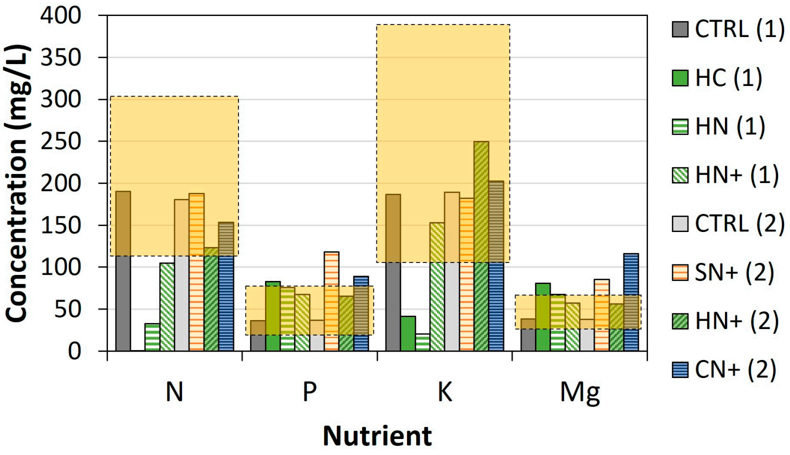

Figure S3. Nutrient concentration in the hydroponic experiments and estimated optimal ranges (±30% values from Table 2). Reference for MgP salts: S, struvite; H, hazenite; C, cattiite. Reference for acids: C, citric acid; N, nitric acid. +, supplemented with KNO_3. In brackets, hydroponic experimental cycle.

3.3.2. Plant Growth Analysis

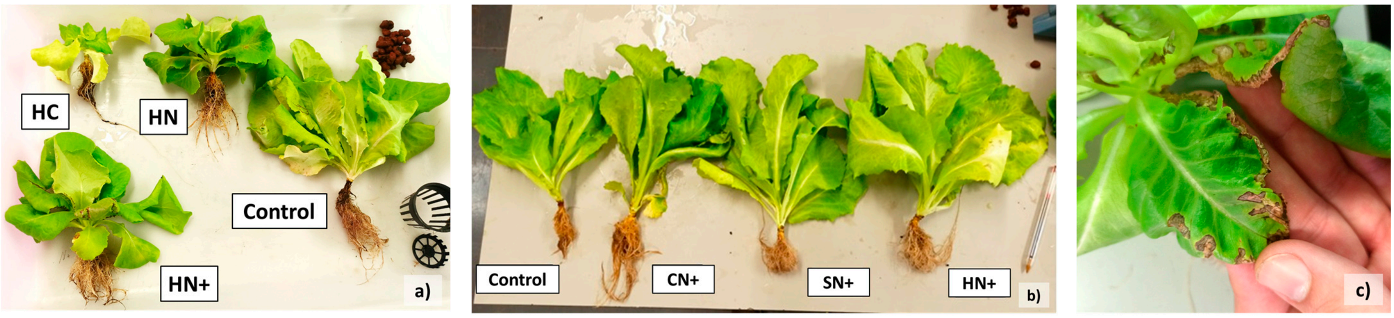

Figure S4. Pictures of the plants after 3 weeks in cycle 1 (**a**) and cycle 2 (**b**), and detail of the tipburn for HN+ condition (**c**).
